# Computationally Efficient Modelling of Stochastic Spatio-Temporal Dynamics in Biomolecular Networks

**DOI:** 10.1038/s41598-018-21826-8

**Published:** 2018-02-22

**Authors:** Jongrae Kim, Mathias Foo, Declan G. Bates

**Affiliations:** 1 School of Mechanical Engineering, University of Leeds, Leeds, LS2 9JT UK; 2 Warwick Integrative Synthetic Biology Centre, School of Engineering, University of Warwick, Coventry, CV4 7AL UK

## Abstract

Measurement techniques in biology are now able to provide data on the trajectories of multiple individual molecules simultaneously, motivating the development of techniques for the stochastic spatio-temporal modelling of biomolecular networks. However, standard approaches based on solving stochastic reaction-diffusion equations are computationally intractable for large-scale networks. We present a novel method for modeling stochastic and spatial dynamics in biomolecular networks using a simple form of the Langevin equation with noisy kinetic constants. Spatial heterogeneity in molecular interactions is decoupled into a set of compartments, where the distribution of molecules in each compartment is idealised as being uniform. The reactions in the network are then modelled by Langevin equations with correcting terms, that account for differences between spatially uniform and spatially non-uniform distributions, and that can be readily estimated from available experimental data. The accuracy and extreme computational efficiency of the approach is demonstrated on a model of the epidermal growth factor receptor network in the human mammary epithelial cell.

## Introduction

Measurement techniques in experimental biology have now advanced to a stage where several molecular species can be tracked at the same time^1,2^. The availability of such data strongly motivates the development of scalable methods for the spatio-temporal modelling of biomolecular networks^3^. It is well known that some characteristics of biomolecular interaction networks are strongly influenced by stochastic noise effects^4–6^. Although the key assumption underlying standard stochastic modelling approaches is spatial homogeneity, molecular interactions in cells are in reality highly spatially heterogeneous^7,8^ and also directionally heterogeneous^9,10^. Current modelling approaches that include both stochastic and spatio-temporal dynamics lead to stochastic reaction-diffusion equations, whose solutions are extremely computationally intensive^11^. This places strong constraints on the size of biomolecular networks that can be modelled using such approaches.

Efficient modelling approaches to take into account directionalities of biomolecules are considered in^9^ and^10^. In this paper, we present a novel method, based on a simple form of the Langevin equation with noisy kinetic constants, to model spatial distribution effects of biomolecular interactions. Spatial heterogeneity in molecular interactions is decoupled into a set of compartments, where the distribution of molecules in each compartment is uniform. Since obtaining such a set of compartments would be challenging in practice, we model the reactions in the network using Langevin equations with correcting terms that account for differences between spatially uniform and spatially non-uniform distributions. We demonstrate the accuracy and computational efficiency of the approach using a model of the epidermal growth factor receptor network in the human mammary epithelial cell.

## Results

### Langevin equation with spatial heterogeneity

Consider the generic model of a ligand-receptor interaction network in a well-mixed solution given by the following five reactions:

1 $$R+L\underset{{k}_{{\rm{off}}}}{\overset{{k}_{{\rm{on}}}}{\rightleftharpoons }}C,\quad C\mathop{\to }\limits^{{k}_{e}}\varnothing ,\quad R\mathop{\to }\limits^{{k}_{t}}\varnothing ,\quad F(t)\to L,\quad {Q}_{R}\to R$$

where *R*, *L* and *C* are the numbers of receptors, ligands and ligand-bound-receptor complex molecules, respectively, *F*(*t*) is the external source of ligand stimulation, *Q*_*R*_ is the intra-cellular receptor generation rate, and *k*_*i*_ for *i* = {on, off, *e*, *t*} is the kinetic rate of each chemical reaction. *k*_on_ is the ligand-receptor binding rate, *k*_off_ is the dissociation rate, and the complex and receptor are internalised with the rates, *k*_*e*_ and *k*_*t*_, respectively. All of the kinetic rates given above are typically assumed to be constant.

The probability that a ligand-bound complex (*C*) is produced from the chemical reaction between the receptor (*R*) and the ligand (*L*) in some small time interval, *dt*, is given by *k*_on_*RLdt*. This is called a propensity function and the propensities for the other five reactions are defined similarly. Now, the probability of the number of receptor molecules being reduced by one, P(*dR* = −1), during the time interval, *dt*, for the well-mixed condition is

2 $${\rm{P}}(dR=-1)={k}_{{\rm{on}}}RLdt+{k}_{t}Rdt$$

where *dR* is the increment in the number of receptors for the time interval, *dt*. Similarly, the probability of the number of receptor molecules increasing by one, P(*dR* = 1), for *dt* is

3 $${\rm{P}}(dR=1)={k}_{{\rm{off}}}Cdt+{Q}_{R}dt$$

The probability that neither of the above two reactions occurs is

4 $${\rm{P}}(dR=0)=[1-{\rm{P}}(dR=-\mathrm{1)}]\,[1-{\rm{P}}(dR=\mathrm{1)}]\approx 1-{k}_{{\rm{on}}}RLdt-{k}_{t}Rdt-{k}_{{\rm{off}}}Cdt-{Q}_{R}dt$$

where terms involving higher orders of *dt* are considered negligible.

**Figure 1:**
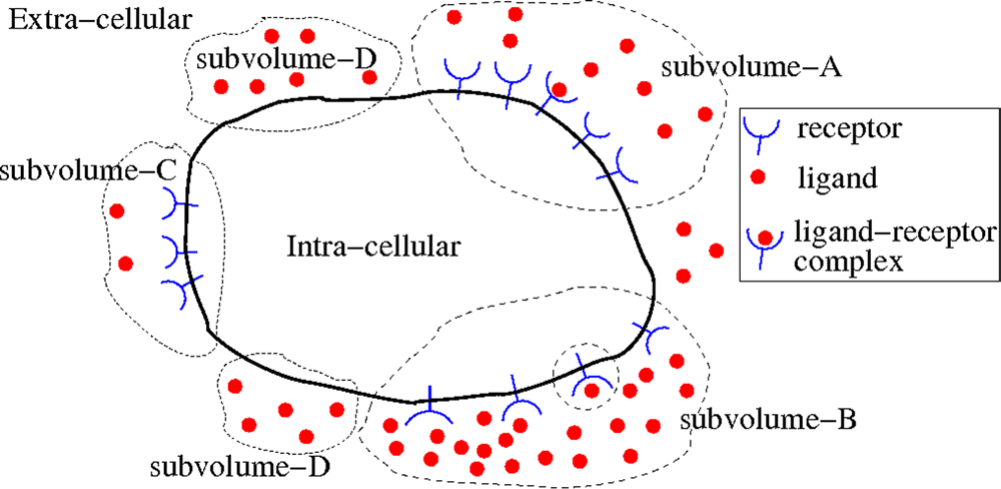
Schematic of receptor-ligand distributions within the cell. The uneven distribution of ligands and receptors can be considered as the sum of multiple subvolumes, where the distributions of each subvolume are approximately uniform. The structure of the subvolumes is not necessarily fixed in time and would change with its own dynamics.

The well-mixed condition considered above is a strong assumption, since it is known that heterogeneous spatial distributions can have significant impact on the responses of biomolecular networks. As illustrated in Fig. 1, the numbers of both receptor and ligand molecules would vary considerably across different regions of the cell surface, and thus the equations derived in (2), (3) and (4) would potentially have large differences from quantities measured by experiments. Typically, the reaction rates, *k*_*i*_, would be measured in a well-mixed condition *in vitro*, or calculated from some theoretical derivations, which most likely neglect spatial effects. To model the effects of spatial heterogeneity while keeping mathematical and computational complexity low, we derive a version of the Langevin equation with spatial fluctuations, as follows.

We assume that the heterogeneous space shown in Fig. 1 can be *virtually* divided into several subvolumes within which the well-mixed condition is valid. Then, the probabilities given above are modified as follows:

5 $${\rm{P}}(dR=-1)={k}_{{\rm{on}}}({R}_{A}{L}_{A}+{R}_{B}{L}_{B}+{R}_{C}{L}_{C}+{R}_{D}{L}_{D})\,dt+{k}_{t}Rdt$$

where *R*_*A*_ and *L*_*A*_ are the number of receptors and ligands in the subvolume-A, the numbers in the other subvolumes are defined similarly, and the total numbers are given by

6a $$R={R}_{A}+{R}_{B}+{R}_{C}+{R}_{D}$$

6b $$L={L}_{A}+{L}_{B}+{L}_{C}+{L}_{D}$$

Note that the kinetic constant, *k*_on_, would be measured in a well-mixed condition *in vitro*. To correct (2) to match with (5), we substitute (6) into the RL term of (2), i.e.,

7 $$\begin{array}{rcl}RL & = & ({R}_{A}+{R}_{B}+{R}_{C}+{R}_{D})\,({L}_{A}+{L}_{B}+{L}_{C}+{L}_{D})\\  & = & ({R}_{A}{L}_{A}+{R}_{B}{L}_{B}+{R}_{C}{L}_{C}+{R}_{D}{L}_{D})\\  &  & +{R}_{A}({L}_{B}+{L}_{C}+{L}_{D})+{R}_{B}({L}_{A}+{L}_{C}+{L}_{D})\\  &  & +{R}_{C}({L}_{A}+{L}_{B}+{L}_{D})+{R}_{D}({L}_{A}+{L}_{B}+{L}_{C})\end{array}$$

Equation (5) is then rewritten using (7) as follows:

8 $${\rm{P}}(dR=-1)={k}_{{\rm{on}}}(1-\delta )RL+{k}_{t}Rdt$$

where the correction term *δ* is given by

9 $$\begin{array}{rcl}\delta  & = & [{R}_{A}({L}_{B}+{L}_{C}+{L}_{D})+{R}_{B}({L}_{A}+{L}_{C}+{L}_{D})+{R}_{C}({L}_{A}+{L}_{B}+{L}_{D})\\  &  & +{R}_{D}({L}_{A}+{L}_{B}+{L}_{C})]/(RL)\end{array}$$

By definition, *δ* is between 0 and 1, where *δ* = 0 corresponds to the well-mixed condition and *δ* = 1 corresponds to a complete separation of the molecules, i.e., no reaction occurs. In most biomolecular networks *δ* would take some value between 0 and 1, and, since the spatial distributions of the molecules might be continuously changing, the value of *δ* would also change over time.

The proposed modelling approach thus provides a compact parameter, *δ*, that captures spatial effects on the stochastic dynamics of a biomolecular network, without requiring the introduction of complex reaction diffusion equations. Note that, in principle, unimolecular reactions would not be affected by spatial heterogeneity, and thus Equation (3) remains the same for both the cases of a well mixed and non-uniform distribution. Only the chemical reactions involving two or more molecules will be affected by spatial heterogeneity.

The expected value of *dR*^2^ is calculated as

10 $$\begin{array}{rcl}{\rm{E}}(d{R}^{2}) & = & {(-\mathrm{1)}}^{2}\,{\rm{P}}(dR=-\mathrm{1)}+{\mathrm{(1)}}^{2}\,{\rm{P}}(dR=\mathrm{1)}\\  & = & {k}_{{\rm{on}}}(1-\delta )RLdt+{k}_{t}Rdt+{k}_{{\rm{off}}}Cdt+{Q}_{R}dt\end{array}$$

and the expected value of *dR* is given by

11 $${\rm{E}}(dR)=-{k}_{{\rm{on}}}\mathrm{(1}-\delta )RLdt-{k}_{t}Rdt+{k}_{{\rm{off}}}Cdt+{Q}_{R}dt$$

where E(·) is the expectation over the sampling space. The variance of *dR* is obtained by

12 $$\begin{array}{rcl}{\rm{E}}\{{[dR-{\rm{E}}(dR)]}^{2}\} & = & {\rm{E}}(d{R}^{2})-{\rm{E}}{(dR)}^{2}\approx {\rm{E}}(d{R}^{2})\\  & = & [{k}_{{\rm{on}}}\mathrm{(1}-\delta )RL+{k}_{t}R+{k}_{{\rm{off}}}C+{Q}_{R}]\,dt\end{array}$$

where all *dt*^2^ terms are negligible. Finally, the Langevin equation is derived by matching the mean and the variance given by (11) and (12), so that

13 $$dR=[-{k}_{{\rm{on}}}\mathrm{(1}-\delta )RL-{k}_{t}R+{k}_{{\rm{off}}}C+{Q}_{R}]\,dt+\sqrt{{k}_{{\rm{on}}}\mathrm{(1}-\delta )RL+{k}_{t}R+{k}_{{\rm{off}}}C+{Q}_{R}}\,d{w}_{R}$$

where *dw*_*R*_ is the Brownian motion with variance equal to $\sqrt{dt}$.

The correction term, *δ*, would have its own stochastic temporal kinetics, for example,

14 $$d\delta ={a}_{\delta }+{b}_{\delta }d{w}_{\delta }$$

where *a*_*δ*_ and *b*_*δ*_ are unknown functions that determine the temporal evolution of *δ*, and *dw*_*δ*_ could be the Brownian motion. In some limited cases, the exact or approximate form of the two functions could be modelled. In general, however, we would use experimental data to identify the structures and/or parameters in the functions determining the temporal evolution of *δ*. For example, if we assume that *a*_*δ*_ and *b*_*δ*_ are constant, then system parameter identification algorithms could be used to determine the constants^12^. This process is described in detail in the following example.

### Example: Epidermal Growth Factor Receptor (EGFR) network in human cells

A model of the ligand-receptor network kinetics of the human mammary epidermal growth factor receptor (EGFR) in the well-mixed state was developed in^13^, where the following set of ordinary differential equations are derived from the underlying chemical reactions, (1), as follows:

15a $$\frac{dR}{dt}=-{k}_{{\rm{on}}}RL+{k}_{{\rm{off}}}C-{k}_{t}R+{Q}_{R}$$

15b $$\frac{dC}{dt}={k}_{{\rm{on}}}RL-{k}_{{\rm{off}}}C-{k}_{e}C$$

15c $$\frac{dL}{dt}=-{k}_{{\rm{on}}}RL+{k}_{{\rm{off}}}C+F(t)$$

where *k*_off_ = 0.24 [1/min], *K*_D_ = 2.47 × 10^−9^ × *N*_av_ × *V*_cell_ [the number of molecules], *N*_av_ = 6.023 × 10^23^, i.e., the Avogadro’s number, ${V}_{{\rm{cell}}}=4\times {10}^{-10}\,[\ell ]$, the cell volume, *k*_on_ = *k*_off_/*K*_D_ *k*_*t*_ = 0.02 [1/min], *Q*_*R*_ = 2 × 10^5^ *k*_*t*_, and *k*_*e*_ = 0.15 [1/min].

**Figure 2:**
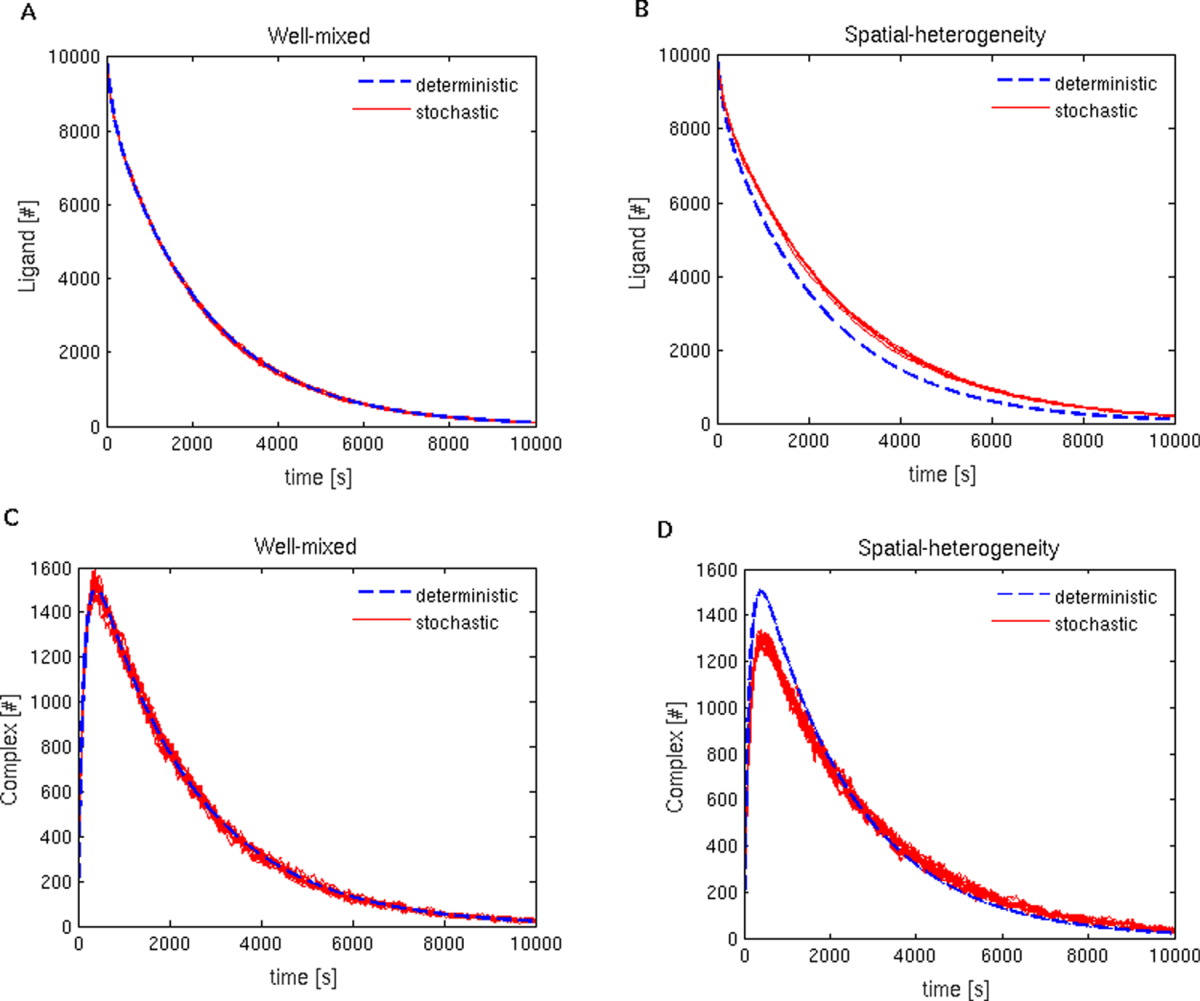
Total number of ligands and complexes in well-mixed and spatially-heterogeneous conditions. For the deterministic case, the time histories of the ligand and complex are obtained by solving the ordinary differential equations corresponding to the molecular interactions. For the well-mixed case, (**A**,**C**) 10 realisations of the exact stochastic simulation, using Gillespie’s direct method, are shown in red lines. (**B**,**D**) 10 realisations of the stochastic reaction-diffusion simulations, shown in red lines, using MesoRD.

Consider an initial impulse input for the ligand, i.e., *L*(0) = 10,000, assuming the presence of 200,000 receptors and no complexes. *F*(*t*) is set to zero, i.e, no external stimulation. The simulation results are shown in Fig. 2. In Fig. 2A,C, stochastic simulations with the well-mixed condition are performed using Gillespie’s direct method, which is an exact stochastic simulation algorithm^14^. As expected, for the well-mixed condition, the 10 stochastic simulations of the model produce trajectories that all closely match the deterministic trajectories produced by solving the ordinary differential equations numerically.

However, even for the case where the well-mixed condition is still valid, the simulations including spatial diffusion produce different results from those generated by the ordinary differential equations or Gillespie’s direct method, as shown in Fig. 2B,D. Here, the ligand and the receptor are uniformly distributed on the surface of the cell, which is assumed to be a sphere of the radius 100 *μ*m. Since Gillespie’s algorithm cannot take into account spatial effects, we solve the reaction-diffusion equations using MesoRD (Mesoscopic Reaction-Diffusion Simulator)^11^, a numerical solver for stochastic reaction diffusion equations using the next subvolume method.

**Figure 3:**
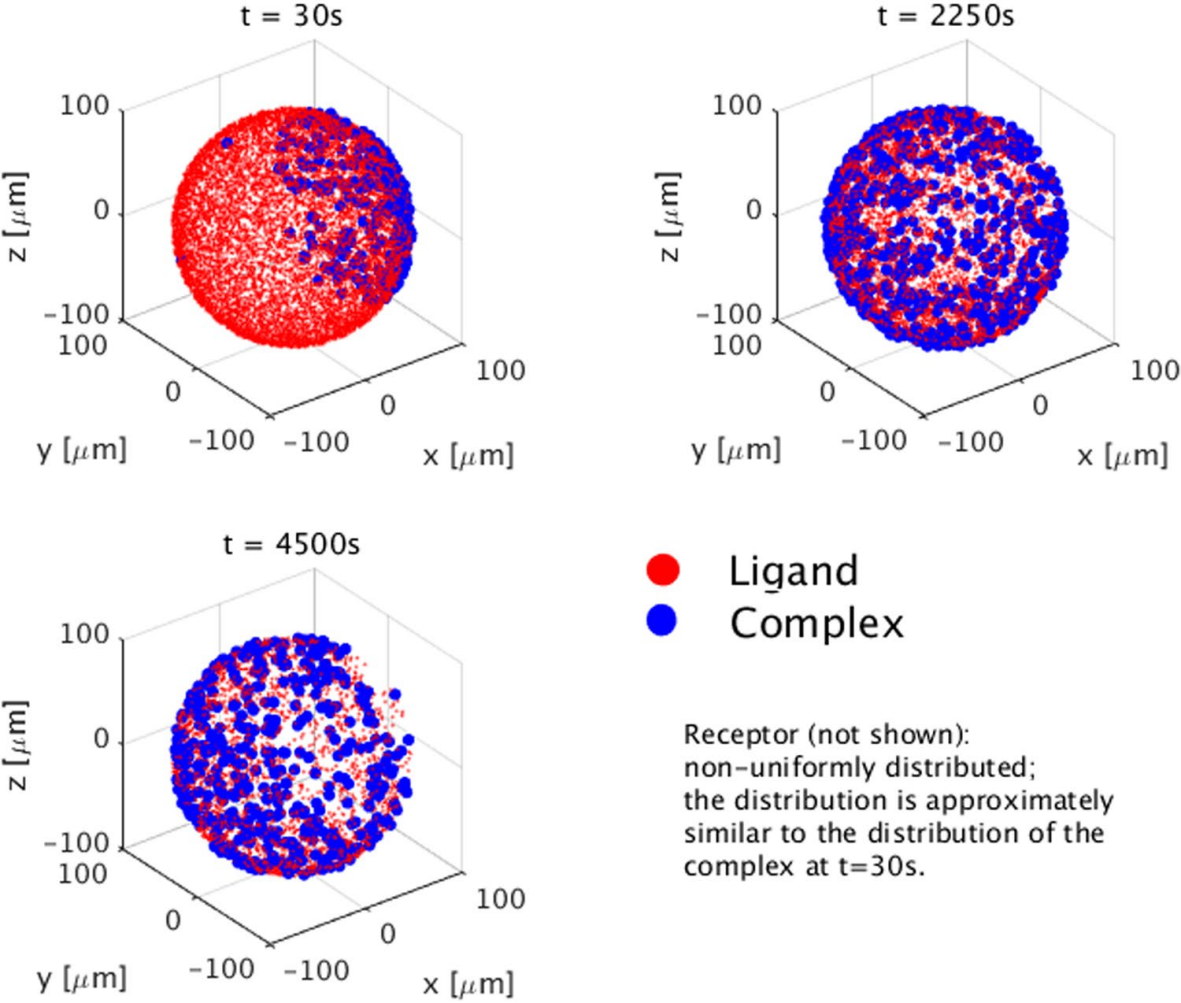
Interaction between non-uniform distribution of receptors and the uniform distribution of ligands.

In reality, it is highly probable that there would be significant differences in the time history of molecular number changes for non-uniform distributions compared to the case of a uniform distribution. To investigate this, a spatially non-uniform distribution is simulated in Fig. 3. Here, the ligands, indicated by the red dots in the figure, are distributed uniformly on the cell surface, while the receptors (not shown in the figure for clarity) are distributed non-uniformly on the cell surface. In this example, we make the distribution of the receptors approximately equal to the distribution of the complexes at *t* = 30 s in the figure, i.e., 99% of the total receptors are placed on the right side of the cell surface and 1% are distributed in the remaining area of the cell surface.

**Figure 4:**
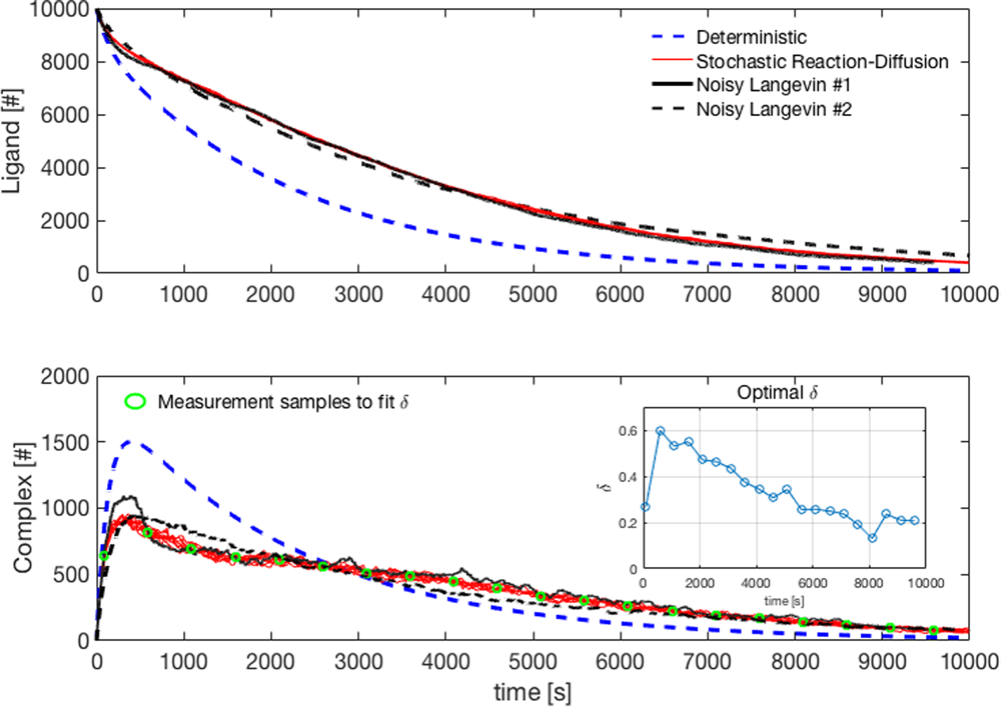
Total number of ligands and complexes in the spatially heterogeneous condition. The time histories of the ligand and the complex obtained from the ordinary differential equations. The 10 stochastic realisations of the reaction-diffusion simulation are carried out using MesoRD and the green circles are the mean values of the MesoRD simulation results (considered as surrogate data points from wet-lab experimental measurements). The new Langevin equation with the optimal *δ* obtained by solving a minimisation problem provides a highly accurate simulation of the molecular time histories in less than one second (Noisy Langevin #1), compared with 38 minutes to compute one reaction-diffusion simulation using MesoRD. The inset shows the corresponding time history of *δ*, which is clearly able to capture the effects of spatial heterogeneity. Less accurate but still reasonable matched trajectories with even lower computational overheads are obtained using a random sampling of *δ* between upper and lower bounds that can be calculated from experimental measurements (Noisy Langevin #2).

The time histories of the ligand and the complex numbers are compared with those calculated by the deterministic simulation in Fig. 4. The dynamic behaviours for both cases are clearly very different, showing that the explicit consideration of spatial heterogeneity is essential in order to correctly capture the dynamics of the molecules in the ligand-receptor network. However, exact simulation algorithms for stochastic reaction diffusion equations are extremely computationally expensive, and hence cannot be applied to larger networks. Moreover, in general, we do not know how the molecules are distributed - in practice we will only be able to measure total concentration changes.

To get around this problem, we use the proposed model based on the Langevin equation with spatial fluctuations, which gives the following stochastic equations:

16a $$dR=[-{k}_{{\rm{on}}}\mathrm{(1}-\delta )RL-{k}_{t}R+{k}_{{\rm{off}}}C+{Q}_{R}]\,dt+\sqrt{{k}_{{\rm{on}}}\mathrm{(1}-\delta )RL+{k}_{t}R+{k}_{{\rm{off}}}C+{Q}_{R}}\,d{w}_{R}$$

16b $$dC=[{k}_{{\rm{on}}}\mathrm{(1}-\delta )RL-{k}_{{\rm{off}}}C-{k}_{e}C]\,dt+\sqrt{{k}_{{\rm{on}}}\mathrm{(1}-\delta )RL+{k}_{{\rm{off}}}C+{k}_{e}C}\,d{w}_{C}$$

16c $$dL=[-{k}_{{\rm{on}}}\mathrm{(1}-\delta )RL+{k}_{{\rm{off}}}C+F(t)]\,dt+\sqrt{{k}_{{\rm{on}}}\mathrm{(1}-\delta )RL+{k}_{{\rm{off}}}C+F(t)}\,d{w}_{L}$$

where the independent parameters *dw*_*R*_, *dw*_*C*_ and *dw*_*L*_ have zero-mean Brownian motion with variance $\sqrt{dt}$, and the dynamics of *δ* depend on the spatial distributions of the molecules involved in the chemical reactions.

In order to obtain a time history of *δ*, assume that a web-lab experiment has been performed and multiple measurements have been obtained of the number of complexes, indicated by green circles in Fig. 4 (here, we use a set of sampled average values from the stochastic reaction-diffusion simulations as surrogate data points). An optimal *δ* for the time period (*t*_*k*−1_, *t*_*k*_] can then be calculated by solving the following minimisation problem:

17 $${\rm{Minimise}}\,J(\delta )=|{\tilde{C}}_{k}-{\bar{C}}_{k}|,$$

where *k* is an integer between 1 and *N*, *N* is the total number of measurements, *t*_0_ is the initial time, ${\tilde{C}}_{k}$ is the complex number measurement at *t*_*k*_, ${\bar{C}}_{k}$ is the mean value of the complex number at *t*_*k*_ provided by solving the noisy Langevin Equation (16), and *dt* is set to 1 s. The minimisation problem is a one-dimensional search, which can be efficiently solved by golden-section search or parabolic interpolation, as implemented in *fminbnd* in the MATLAB optimisation toolbox^15^. The results are shown in Fig. 4. As expected from the stochastic spatial-diffusion simulation setting, the spatial heterogeneity is larger at the beginning and slowly diminishes as the molecules interact and are diffused. The Langevin equations are integrated using the 1st-order Euler method. The comparisons with the trajectories from the solutions of the full 3-dimensional reaction diffusion equations are shown in Fig. 4, where both the number of ligands and the number of complexes (Noisy Langevin #1 in the figure) are well matched with the spatial-diffusion simulation. As shown in the figure, the proposed modelling approach provides a much better approximation of the true network dynamics than the ordinary differential equation based model, and in fact closely approximates the exact solution provided by solving the reaction diffusion equations.

Crucially, however, solving the noisy Langevin equations requires only a fraction of the computing time - less than a second to solve one complete time history for this example on a typical desktop computer. On the other hand, it takes about 38 minutes for MesoRD to finish one simulation^16^. On the same computer, for the uniform mixed condition, MCell, another Monte-Carlo stochastic reaction-diffusion simulator, takes about 24 minutes to obtain one realisation of the stochastic simulation^17^.

Note that a simpler approach of estimating *δ* instead of solving the optimisation problem would also be possible, and could potentially be useful for application of the approach to large-scale networks. Here, we assume that *δ* is a uniform distribution over $[\underline{u},\bar{u}]$ which changes every *t*_*N*_ sampling time, and $\underline{u}$ and $\bar{u}$ are estimated using the total concentration changes from the experimental measurements. The number of complex molecules is then calculated for various values of $\underline{u}$ and $\bar{u}$ to fit the measurements. and *t*_*N*_ is set to 10 s. *t*_*N*_ is set to a value greater than *dt* as spatial fluctuations would not be faster than the chemical reactions. For our example, the estimated bounds for the best fit are $\underline{u}=0.3$ and $\bar{u}=0.7$. As expected, the resulting simulations are less accurate than those generated by the optimisation based approach, but reasonably well matched trajectories (Noisy Langevin #2 in Fig. 4) are still obtained.

## Conclusions

A novel modelling methodology for simultaneously capturing stochastic and spatial effects on the dynamics of biomolecular networks was presented. We showed how, using a relatively simple formalism, i.e. the Langevin equation with noisy kinetic constants, spatial heterogeneity in the interaction space of the network can be included in the model. The effectiveness of the modelling approach is shown through its application to the modelling of the epidermal growth factor receptor network of a human mammary epithelial cell. Using the proposed method, highly accurate simulations of stochastic spatio-temporal dynamics were produced with massively reduced computational overheads. As spatial heterogeneity produces noticeably different dynamics in the network, it is vital that future modelling formalisms are able to take this heterogeneity into account, while minimising the computational complexity of the resulting simulations. The method presented in this paper represents a significant step forward in achieving this goal.

## Methods

### Stochastic Simulations

For the well-mixed cases, the Gillespie’s direct method is implemented and the simulations are performed using MATLAB^18^. The reaction-diffusion equations are solved using MesoRD Ver. 1.1^11^. The uniform mixed condition was also solved using MCell for the comparison of the computation time^17^. The noisy Langevin equation is implemented in MATLAB and solved using the 1st-order Euler method with the integration interval equal to 1 seconds.

### Computer Hardware

All simulations are performed in a desktop computer with the CentOS Release 6.9, Intel Xeon Quadprocessor 3.20 GHz with memory of 15.6 GB.

### Data availability

No datasets were generated or analysed during the current study.

## References

[1] Roeffaers MBJ (2007). Single-molecules fluorescence spectroscopy in (bio)catalysis. Proc. Natl. Acad. Sci..

[2] Wen J-D (2008). Following translation by single ribosomes one codon at a time. Nature..

[3] van Zon JS, Morelli MJ, Tanase-Nicola S, ten Wolde PR (2006). Diffusion of transcription factors can drastically enhance the noice in gene expression. Biophys. J..

[4] Elowitz MB, Levine AJ, Siggia ED, Swain PS (2002). Stochastic gene expression in a single cell. Science..

[5] Vilar JMG, Kueh HY, Barkai N, Leibler S (2002). Mechanisms of noise-resistance in genetic oscillators. Proc. Natl. Acad. Sci..

[6] Pedraza JM, van Oudenaarden A (2005). Noise propagation in gene networks. Nature..

[7] Ventura BD, Lemerle C, Michalodimitrakis K, Serrano L (2006). From *in vivo* to *in silico* biology and back. Nature..

[8] Fange D, Elf J (2006). Noise-induced Min phenotypes in *E*. *coli*. PLoS Comput. Biol..

[9] Yang J (2008). Kinetic Monte Carlo Method for Rule-Based Modeling of Biochemical Networks. Phys. Rev. E.

[10] Ruiz-Herrero T, Estrada J, Guantes R, Miguez DG (2013). A Tunable Coarse-Grained Model for Ligand-Receptor Interaction. PLoS Comput. Biol..

[11] Hattne J, Fange D, Elf J (2005). Stochastic reaction-diffusion simulation with MesoRD. Bioinformatics..

[12] Kim J, Bates DG, Postlethwaite I, Heslop-Harrison P, Cho K-H (2007). Least squares methods for identifying biochemical regulatory networks from noisy measurements. BMC Bioinformatics..

[13] Shankaran H, Resat H, Wiley HS (2007). Cell surface receptors for signal transduction and ligand transport: A design principles study. PLoS Comput. Biol..

[14] Gillespie DT (1977). Exact stochastic simulation of coupled chemical reactions. J. Physc. Chem..

[15] MATLAB Optimization Toolbox version 7.6 (R2017a) The MathWorks Inc., Natick, Massachusetts (2017).

[16] Fange D, Mahmutovic A, Elf J (2005). MesoRD 1.0: Stochastic reaction-diffusion simulations in the microscopic limit. Bioinformatics..

[17] Bartol TM, Dittrich M, Faeder JR (2005). MCell. Encyclopedia of Computational Neuroscience.

[18] MATLAB version 9.2.0. (R2017a) The MathWorks Inc., Natick, Massachusetts (2017).

